# Cognitive load gating system in motor imagery BCIs: a dual-task EEG study with differential entropy-based reliability estimation

**DOI:** 10.3389/frai.2026.1859963

**Published:** 2026-07-13

**Authors:** Hamrita Haridharan, Gomathy Dhanasekar, Sharmila Nageswaran

**Affiliations:** School of Electronics Engineering, Vellore Institute of Technology, Vellore, India

**Keywords:** brain-computer interface, cognitive load, differential entropy, dual task, EEG, FBCSP, motor imagery, Riemannian geometry

## Abstract

Brain–computer interface (BCI) systems based on motor imagery hold significant clinical value for individuals who have lost voluntary movement, but most studies test BCI assistive devices like powered wheelchairs under ideal conditions where motor imagery signals are not interfered by simultaneous cognitive load. This work records electroencephalography (EEG) from 13 participants across four tasks: baseline rest, mental arithmetic (Easy, Medium, Hard), pure left/right motor imagery and both tasks simultaneously, to build a two-layer classification system. The first layer decodes motor intent using a model chosen from nine classifiers, from Filter Bank Common Spatial Pattern (FBCSP) and Riemannian geometry families. The second layer is a subject-specific safety gate that combines the classifier’s decision-margin confidence score with 39-dimensional Differential Entropy (DE) features extracted from the theta (4–8 Hz), alpha (8–13 Hz), and beta (13–30 Hz) frequency bands across all 13 electrodes, feeding a logistic regression boundary to predict trial-level MI prediction reliability. FBCSP + SVM-Linear model emerged as the best-performing model on pure motor imagery cross-validation. Under 10-fold cross-validated pure motor imagery, the mean balanced accuracy across all subjects was 0.594 and degraded to 0.517 on dual-task trials, a statistically significant reduction (Wilcoxon W = 14.0, *p* = 0.026). The DE-based safety gate, operating at a mean rejection rate of 25.0%, produced statistically significant reductions in false positive commands (from 8.00 to 5.62 per subject, *p* < 0.001) and false negative commands (from 13.00 to 9.00 per subject, *p* < 0.001). Post-gate balanced accuracy improved significantly (*p* = 0.013). An ablation study showed that DE features drive gate performance. These results demonstrate that a learned cognitive load gate has the potential to improve the safety profile of a motor imagery BCI in a preliminary proof-of-concept offline evaluation with healthy participants.

## 1 Introduction

Motor imagery based Brain–Computer interfaces (MI-BCIs) offer a pathway to non-muscular communication and environmental control for individuals with motor impairments, like those affected by amyotrophic lateral sclerosis, spinal cord injury, and brainstem stroke (Padfield et al., 2019; Saibene et al., 2023). These systems decode imagined movement using scalp EEG by detecting Event-Related Desynchronization (ERD) and Event-Related Synchronization (ERS) within the mu (8–13 Hz) and beta (13–30 Hz) sensorimotor rhythms, which exhibit characteristic power reductions during motor imagination (Pfurtscheller and Neuper, 2001). The spatial and temporal properties of ERD and ERS have been characterized extensively, with lateralised beta rebound following the end of a movement, a consistent and reproducible marker in both executed and imagined movements (Pfurtscheller, 2000). Powered wheelchair control is where MI-BCI research has its most clinically significant application. Recent work has confirmed that directional control is achievable in structured indoor settings (Palumbo et al., 2021; Choi et al., 2023). Theta power between 4–8 Hz rises reliably with working memory demand, and alpha suppression across 8–12 Hz tracks attentional engagement during tasks like mental arithmetic (Onton et al., 2005; Klimesch, 1999). Both phenomena are now standard in the passive BCI literature.

Yet the MI-BCI wheelchair literature has a gap that has persisted for over a decade. Nearly all such systems are tested under single-task conditions where the user imagines a movement with their full attention which bears no resemblance to actual wheelchair operation. Navigating a real environment requires simultaneous spatial reasoning, hazard awareness and divided attention (Palumbo et al., 2021; Naser and Bhattacharya, 2023). Second, although cognitive load monitoring from EEG is well studied in passive BCI contexts, it has rarely been integrated with active MI decoding within the same recording session (Hussain et al., 2025). Third, existing BCI safety mechanisms typically rely on fixed decision thresholds or post-hoc error detection instead of trial-level reliability estimation, making them poorly suited to the time-varying cognitive demands of real navigation (Pan et al., 2024). This results in a system that performs well when the user is fully attentive but does not safeguard against the erroneous commands that cognitive interference introduces, a problem given that a single misclassified command in a powered wheelchair could harm the user’s physical safety.

This work addresses these gaps by proposing and evaluating a two-layer system. The first layer selects and applies the best-performing MI decoder from nine candidate pipelines. The second layer is a subject-specific safety gate that learns, from each individual’s dual-task data, whether a given MI prediction is reliable enough to be passed to the actuator. The gating signal is constructed from DE features across the theta, alpha, and beta bands computed over all 13 electrodes, combined with the MI decoder’s decision-margin confidence score. A structured four-part recording protocol was administered to 13 participants using a clinical-grade EEG system, encompassing baseline rest, graded mental arithmetic, pure motor imagery, and simultaneous dual-task trials. A logistic regression meta-classifier defines a learned acceptance boundary in the DE-confidence feature space for each subject, replacing manual threshold tuning in an offline, proof-of-concept evaluation. Wilcoxon signed-rank tests confirm statistically significant reductions in both false positive and false negative command errors post-gate.

The contributions of this work are: (1) a dual-task EEG recording protocol that measures how much MI decoder accuracy falls under graded cognitive load, with a group-level Wilcoxon test giving W = 14.0, *p* = 0.026; (2) a per-subject binomial test to interpret all gate results; (3) a 39-dimensional Differential Entropy feature space extracted from all 13 electrodes across three frequency bands; (4) a subject-specific logistic regression safety gate that produces statistically significant FN reductions beyond random rejection (*p* = 0.006), with FP reductions that are significant against the pre-gate baseline but not against matched random rejection; and (5) an ablation study showing that DE features are the main driver of gate performance, not the SVM confidence score.

## 2 Literature review

### 2.1 Challenges with motor imagery BCIs

Motor imagery-based BCIs exploit the sensorimotor rhythms generated during imagined movement to route control signals to external devices without muscle involvement. Patients who lack voluntary movement due to conditions such as locked-in syndrome and high spinal cord injury represent the primary target user group. Two decades of work have refined MI-BCI-driven wheelchairs from simulated tracks to real indoor navigation (Palumbo et al., 2021; Choi et al., 2023). Translating this into assistive use exposes the gap where the user’s cognitive state at any given moment interferes with the EEG signal. The effect of fatigue, frustration, and attentional state on decoder output was observed by Myrden and Chau across 12 participants (Myrden and Chau, 2015). Self-reported mental state was a statistically significant predictor of classification accuracy with fatigue specifically producing quantifiable drops in decoding quality. A classifier calibrated on a rested, attentive user will not behave the same way when that user is fatigued, distracted, or cognitively occupied with navigating a route.

### 2.2 Dual-task interference

Frontal theta and parietal alpha perturbations associated with mental effort overlap spectrally and spatially with the sensorimotor rhythms that MI decoders depend on. Edelman et al. (2018) demonstrated that users can simultaneously operate a sensorimotor rhythm BCI alongside a second paradigm, but the cognitive demands of managing two control streams introduced performance variability that a single-task evaluation did not. Käthner et al. (2014) recorded alpha and theta band power alongside P300 amplitudes while participants operated an ERP-based BCI under increasing mental workload and fatigue and found that both alpha power and P300 amplitude were sensitive to these states during BCI use. When a user’s cognitive resources are divided, the MI signal degrades in a way that produces erroneous or absent commands, and standard decoders have no mechanism to distinguish a poor-quality prediction from a reliable one.

### 2.3 EEG markers of cognitive load

Frontal theta power (4–8 Hz) rises with working memory demand, while parietal alpha (8–12 Hz) suppresses when attentional resources are being utilized. Klimesch’s review showed the theta-alpha coupling is not task-specific. It holds whether subjects are doing arithmetic, navigating spatial layouts, or retrieving memories (Klimesch, 1999). Puma et al. (2018) pushed this further: 20 participants managed one to four overlapping sub-tasks simultaneously while the researchers tracked EEG power, NASA-TLX ratings, and pupil diameter together. Both bands scaled up with sub-task count, but flattened once accuracy started to slip, indicating the limits of neural resource allocation. Graded mental arithmetic was used in this study rather than other load manipulations because it is the most experimentally controllable option available. The dose-dependent theta response to arithmetic difficulty has now shown up in enough independent labs to be considered reliable (Lim et al., 2018).

### 2.4 BCI illiteracy and sparse montages

CSP filters work better with more electrodes due to their spatial filtering. As electrode count drops, the filters become less selective, and accuracy drops with them (Lotte et al., 2018). Somewhere between 15 and 30% of BCI-naive users cannot generate EEG patterns distinguishable enough for any decoder, regardless of how much additional training they receive, called BCI illiteracy (Sannelli et al., 2019). Subject-independent approaches have been proposed to reduce calibration burden, though they have not fully resolved the illiteracy problem in naive or disabled populations (Kwon et al., 2020). On sparse montages with untrained participants, two-class MI balanced accuracy is typically between 0.55 and 0.65, well below the 0.70–0.80 figures from competition datasets where participants trained for weeks with dense caps (Lotte et al., 2018). All 13 subjects in this study had no prior BCI experience and that places the expected accuracy range squarely in the moderate zone. Subjects with near-chance performance were retained in every downstream analysis because the gate’s whole purpose is to protect exactly those users.

### 2.5 Feature extraction and MI classifiers

FBCSP and Riemannian geometry are two families that have consistently outperformed alternatives on MI tasks. FBCSP runs CSP spatial filtering across several frequency sub-bands to extract their log-variance features, capturing band-specific lateral ERD/ERS (Ang et al., 2012). Müller-Gerking et al. (1999) formalized the single-trial CSP approach that became the field’s standard spatial filter for mu and beta band decoding. Joint optimization of time, frequency, and spatial dimensions has shown gains over standard CSP on multi-session MI data, underscoring that the feature space assumptions of single-domain filters are a practical bottleneck (Miao et al., 2021). Riemannian methods take a different route entirely where EEG covariance matrices are treated as points on the manifold of symmetric positive-definite matrices, and classification is done by geodesic distance rather than Euclidean distance. Barachant et al. (2012) demonstrated on BCI Competition IV Dataset IIa that the MDM classifier matched CSP + LDA in accuracy, and that projecting onto the tangent space before LDA lifted the mean from 65.1 to 70.2%. On small datasets especially, Riemannian methods are more practical because they have fewer free parameters and make weaker stationarity assumptions than CSP-based pipelines (Congedo et al., 2017). Recent work on unilateral limb decoding has reinforced that Riemannian-based classifiers generalize more reliably than filter-based methods when the training data is limited or the target population is heterogeneous (Rong et al., 2024). Nine pipelines comprising FBCSP and Riemannian methods were benchmarked and the final model was selected based on the performance.

### 2.6 Safety gating and cognitive load monitoring

Frontal theta is the most reliably observed EEG marker of working memory load across mental arithmetic, n-back, and spatial tasks (Onton et al., 2005; Klimesch, 1999). Mastropietro et al. (2023) showed that workload indices derived from EEG remain stable across electrode configurations as long as frontal coverage is preserved, supporting the use of sparse montages for monitoring the cognitive state as long as the frontal coverage is maintained. Hussain et al. (2025) built a mental-state-aware BCI wheelchair system that monitors EEG band power in real time and triggers a contingency response whenever detected stress crosses a preset value, reaching 74.26% mental state prediction accuracy, although a meaningful result, the threshold was set in advance and applied identically to every user. The system in this study learns a separate decision boundary for each individual from their own dual-task session data, which is a substantively different design choice. Saeedi et al. (2017) showed that online prediction of command reliability, used to modulate shared control assistance for a tetraplegic user over an 11-month study, produced more stable long-term BCI performance than a fixed-threshold approach, and that accepting a modest reduction in command throughput in exchange for improved reliability was a clinically sound trade-off. Zander and Kothe (2011) introduced the broader conceptual framework for this kind of system, proposing that spontaneously generated brain signals reflecting cognitive and affective user state could be monitored passively to make human-machine systems neuroadaptive rather than unresponsive to the user’s changing mental condition.

Differential Entropy (DE) was selected as the cognitive load feature. For a Gaussian-distributed band-limited signal, DE is mathematically equivalent to log band power but expressed in an information-theoretic framework that is more principled for cross-subject and cross-band comparison. Duan et al. (2013) showed that DE features outperform energy spectrum features for EEG-based classification, with average accuracy of 84.22% compared to 76.56% on emotion recognition tasks. Zhang and Min (2020) separately demonstrated 87.3% accuracy in four-class emotion classification using DE features extracted with wavelet packet decomposition and random forest classification, illustrating DE’s sensitivity to subtle spectral power differences across cognitive states. Zanetti et al. (2022) confirmed that EEG-based cognitive workload monitoring using spectral features on as few as four peripheral channels can achieve 74.5% accuracy on wearable hardware in real time, supporting the practical viability of the DE-based approach on sparse montages.

## 3 Methodology

### 3.1 Participants

Thirteen healthy adult volunteers were recruited from Vellore Institute of Technology (7 male, 6 female; mean age 20.54 ± 2.4 years, range 17–27). All participants were right-handed and reported no history of neurological or psychiatric disorders. None had any prior experience with brain-computer interfaces.

### 3.2 Data acquisition

EEG was recorded using a Clarity BrainTech clinical-grade amplifier at 256 Hz, stored in European Data Format (EDF). Thirteen scalp electrodes (Fp1, Fp2, F3, Fz, F4, C3, Cz, C4, P3, Pz, P4, O1, O2) were positioned according to the international 10–20 system and referenced to a common REF electrode. These channels provide bilateral fronto-central-parieto-occipital coverage spanning both the sensorimotor strip relevant to hand motor imagery ERD/ERS (Pfurtscheller and Neuper, 2001) and the frontal and occipital regions associated with cognitive load modulation (Onton et al., 2005; Klimesch, 1999).

The experimental protocol comprised four parts. Part 1 was 60 s of eyes-open rest where participants were simply told to relax and look at a blank screen. Part 2 had 20 mental arithmetic trials, split across three difficulty levels: Easy (single-digit addition or subtraction), Medium (two-digit addition), and Hard (two-digit subtraction and multiplication). Part 3 consisted of 40 pure motor imagery trials, 20 left-hand and 20 right-hand, in random order. Part 4 had 40 dual-task trials where the subject had to do both simultaneously, with matched arithmetic difficulty. Each active phase lasted 4 s, preceded by a 2-s fixation cross and followed by 2 to 3 s of blank screen. Each part was separated by a 10-s break.

### 3.3 Preprocessing

A 50 Hz notch filter was used to suppress the power-line interference, followed by a first-order IIR Butterworth bandpass filter from 1–40 Hz. After that, Independent Component Analysis (FastICA) was applied across all 13 channels to remove the ocular artefacts. Fp1 and Fp2 served as EOG proxy channels with a correlation threshold of 3.0 standard deviations. Component-based decomposition of EEG, whether for artefact rejection as with ICA, or for signal-to-noise enhancement as with task-related component analysis in SSVEP decoding (Nakanishi et al., 2018), has proven useful across BCI paradigms beyond motor imagery. At most one independent component was removed per subject. Of 13 subjects, 8 had one EOG component removed (S02, S03, S05, S06, S07, S08, S12, S13); 5 had no component identified above threshold (S01, S04, S09, S10, S11) and their signals were left unchanged. This conservative, subject-specific approach removes only the most salient blink artefact while preserving all neural signal. Fp1 and Fp2 were retained in the data after ICA for subsequent DE feature extraction, where frontal channels contribute the largest cognitive load signatures. All 40 trials per part were retained for every subject, yielding perfectly balanced Left/Right class distributions (20 trials per class) throughout.

Epochs were extracted from 0.5 to 4.0 s relative to each Active marker onset, yielding 3.5 s of analysis data per trial. The 0.5-s onset buffer discards the visual evoked potential generated by reading the cue text, which would otherwise contaminate covariance matrices with a non-motor neural transient (Blankertz et al., 2008).

### 3.4 Feature extraction: motor imagery

**Figure 1:**
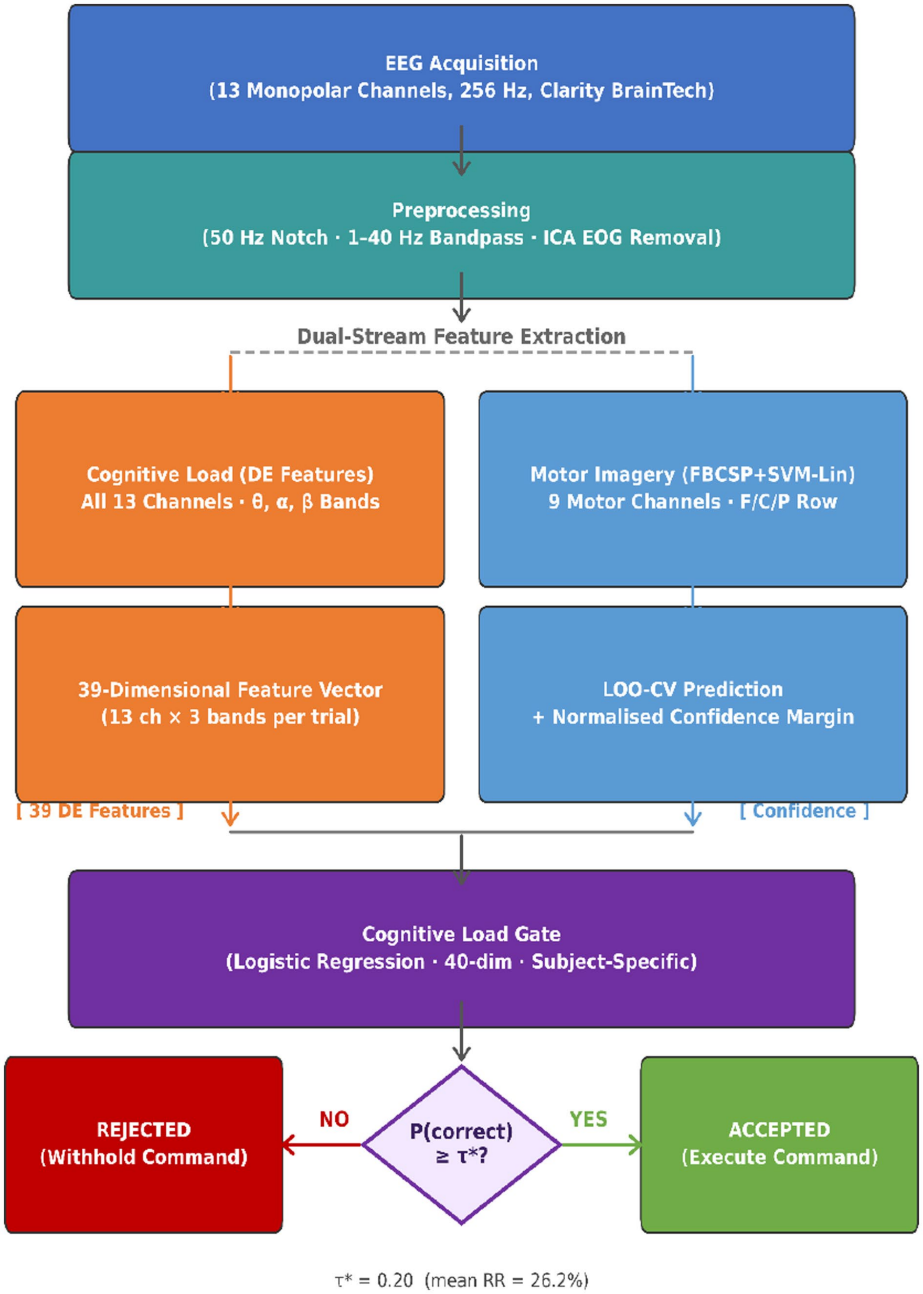
Proposed dual-stream BCI architecture. EEG is preprocessed and fed to the motor imagery decoder (FBCSP with linear-kernel SVM) and the cognitive load feature extractor (39-dimensional DE). A subject-specific LR gate combines the decoder’s normalized confidence margin with DE features to determine whether each predicted command is passed to the wheelchair actuator or withheld.

Nine classifier pipelines were evaluated on pure Part 3 MI trials to identify the best-performing model, following two parallel feature extraction strategies on the 9 motor-relevant channels (F3, Fz, F4, C3, Cz, C4, P3, Pz, P4) as shown in Figure 1.

FBCSP family: Epochs were bandpass-filtered into mu (8–13 Hz), low-beta (13–20 Hz), and high-beta (20–30 Hz) sub-bands using zero-phase fourth-order Butterworth filters. CSP spatial filters were fitted on the training partition for each sub-band with Ledoit-Wolf regularisation (Ledoit and Wolf, 2004), and four components were extracted per band. Log-variance features were computed from the projected signals, resulting in a 12-dimensional feature vector per window (4 components × 3 bands). Each trial was segmented into overlapping 1-s windows with 0.5-s step, generating approximately 6 windows per trial (Gaur et al., 2021). Window-level predictions were aggregated by majority vote to yield a single trial-level prediction. Four classifiers were paired with FBCSP features: linear discriminant analysis (LDA), logistic regression (LR), support vector machine with a linear kernel (SVM-Linear), and SVM with a radial basis function kernel (SVM-RBF).

Riemannian family: Full-trial covariance matrices were estimated from broadband (1–40 Hz) epochs of the 9 motor channels using Ledoit-Wolf shrinkage regularisation. No sliding window was applied and the full 3.5-s epoch was used to maximize SPD matrix stability (Barachant et al., 2012). Five classifiers were evaluated: Minimum Distance to Riemannian Mean (MDM), TSM + LDA, TSM + LR, TSM + SVM-Linear, and TSM + SVM-RBF. MDM assigns each test covariance to the class whose Riemannian mean is geodesically nearest, providing parameter-free deterministic classification (Barachant et al., 2012). Tangent Space Mapping (TSM) linearises the manifold locally, allowing standard Euclidean classifiers to operate on covariance data (Congedo et al., 2017).

All nine pipelines were evaluated under 10-fold stratified k-fold cross-validation. Trial indices were used as the stratification variable, preventing windows from the same trial sharing an identical class label from appearing in both the training and testing sets simultaneously, a leakage pathway that would otherwise allow the classifier to memorize patterns within a trial and return artificially inflated accuracy values (Schirrmeister et al., 2017). With 40 balanced trials per subject, each 10-fold split gave 36 trials for training and 4 for testing which is adequate for the covariance-based Riemannian methods. Two selection criteria were used in parallel. Criterion A was 10-fold stratified balanced accuracy on the Part 3 pure MI trials, measuring how well each model decodes uncontaminated imagery and criterion B was trained on all of Part 3 and tested on all of Part 4. This is a cross-condition transfer test measuring whether MI decoding survives the interference of cognitive load. The model with the highest Criterion A mean across all subjects was chosen.

### 3.5 Feature extraction: cognitive load

Differential Entropy features were extracted from Part 4 dual-task epochs using all 13 channels. For each trial, each channel was independently bandpass-filtered into three bands: theta (4–8 Hz), alpha (8–13 Hz), and beta (13–30 Hz) using zero-phase fourth-order Butterworth filters. DE was then computed as shown in Equation 1.


$$\mathrm{DE}=0.5\times \log(2\mathrm{\pi e}\times \sigma^{2}+\varepsilon )$$ (1)

where σ^2^ is the variance of the band-filtered signal in μV^2^, and the ε = 10^−8^ term is a small constant added to avoid numerical instability. Raw EEG data were converted from MNE-internal units (Volts) to μV before variance computation. This yields a 39-dimensional feature vector per trial (13 channels × 3 bands). Computing DE independently per channel preserves spatial information about load-related spectral shifts: frontal channels (Fp1, Fp2, F3, Fz, F4) capture frontal theta elevation under arithmetic load (Onton et al., 2005); occipital channels (O1, O2) capture alpha suppression under attentional engagement (Klimesch, 1999); and motor channels (C3, Cz, C4, P3, Pz, P4) capture sensorimotor band DE changes arising from the interaction between MI and concurrent arithmetic (Duan et al., 2013).

Computing DE per channel, rather than averaging across electrode groups, was a deliberate choice rooted in how cognitive load actually behaves in the EEG. The effect is not uniform across the scalp. Frontal theta elevation under arithmetic is concentrated at Fp1, Fp2, F3, Fz, and F4, because those sites sit over the prefrontal working memory network. Alpha suppression under attentional engagement is strongest at the parietal and occipital sites P3, Pz, P4, O1, and O2. Beta-band disruption from the motor-arithmetic overlap is most visible at C3, Cz, and C4. Averaging across channels before feeding the gate would discard all of this spatial structure. The logistic regression assigns a separate weight to each of the 39 dimensions independently per subject, so it can put high weight on the frontal theta channels that are most load-sensitive for that person and down-weight the ones that carry noise. Keeping the per-channel representation is what makes the gate’s learned boundary neurophysiologically meaningful and subject-specific.

### 3.6 Meta-classifier safety gate

The safety gate runs on Part 4 dual-task trials. Two inputs are combined into a single feature vector for each trial: the 39-dimensional DE vector from Section III.5, and the MI decoder’s normalized decision-margin confidence score. Confidence was taken as the absolute difference between the predicted class probability and the alternative class probability from the SVM’s calibrated output, then scaled to the range [0, 1] across all Part 4 trials within each subject. This produces a 40-dimensional input vector per trial.

A subject-specific logistic regression meta-classifier was trained in this 40-dimensional space. Class-weight balancing was applied to handle the unequal split between correct and incorrect predictions that typically occurs within a single subject’s dual-task data. All 40 features were scaled to zero mean and unit variance before fitting.

A strictly nested cross-validation scheme was applied, using leave-one-out as the outer fold structure, to ensure fully out-of-fold performance estimates with no data leakage between gate training, threshold selection, and evaluation. For each of the 40 Part 4 trials, the gate was trained on the other 39. The acceptance threshold was also chosen on those same 39 trials by sweeping values from 0.10 to 0.90 in steps of 0.05 and picking the one that maximized combined FP and FN reduction while keeping the rejection rate within the 20 to 30 percent window. The held-out trial was then accepted or rejected using that fold-specific threshold.

To check whether the gate’s error reductions were simply a result of issuing fewer commands rather than selective suppression of unreliable trials, a permutation-based random rejection baseline was computed for each subject. At each subject’s actual gate rejection rate, 1,000 random subsets of that same size were drawn from the available trials and the mean FP and FN reductions were recorded across permutations. The gate’s per-subject reductions were then compared against this null distribution in two ways: at the individual level, using a one-sided 95th percentile cutoff, and at the group level, using a two-tailed Wilcoxon signed-rank test that compared each subject’s learned gate reduction against that subject’s random baseline mean.

The 20 to 30 percent rejection rate constraint follows the operational framework of Ferrez and Millán (2008), who showed that this range gives meaningful error suppression without cutting command throughput to an unworkable level. Trials where the gate’s output probability exceeds the accepted threshold are passed to the actuator. All others are withheld.

### 3.7 Evaluation metrics

Balanced accuracy is the primary metric used here. It is the average of per-class recall and does not get inflated when class sizes are unequal, which matters here because the gate changes how many trials from each class get accepted (Lotte et al., 2018). In this study, LEFT is the positive class (label 0). A false positive means the decoder predicted LEFT when the actual label was RIGHT. A false negative means the decoder predicted RIGHT when the actual label was LEFT. Both types of error are physically dangerous in wheelchair control (Palumbo et al., 2021).

To check whether each subject’s decoder was operating above chance, a one-sided binomial test was applied to both Criterion A and Criterion B balanced accuracy scores. Each test used n = 40 trials, with the null hypothesis that balanced accuracy is at most 0.5, and *p* < 0.05 as the cutoff. All FP and FN values were recomputed directly from each subject’s confusion matrix to make sure the numbers are internally consistent. Recall was computed as TP/(TP + FN) and specificity as TN/(TN + FP), each from the confusion counts directly.

Pre-gate and post-gate values were compared using two-tailed Wilcoxon signed-rank tests across all 13 subjects (Wilcoxon, 1945). When recall and specificity come out numerically equal for a subject, it is because FP equals FN under balanced classes, which is a mathematical outcome of the class structure, not a reporting mistake. The significance level used throughout was *p* < 0.05.

### 3.8 Ablation analysis

Three gate variants were tested under the same nested LOO scheme to check whether both inputs actually contribute to gate performance. The first variant used only the 1-dimensional normalized SVM confidence score (Confidence-only). The second used only the 39-dimensional DE feature vector with no confidence input (DE-only). The third was the full 40-dimensional combined gate. Training, threshold selection, and test evaluation were separated fold by fold in all three variants. If the DE features do not provide statistically significant improvement over the confidence-only gate, the gate’s contribution should be attributed to the DE feature set alone and the confidence input framed as supplementary.

A channel subset comparison was also carried out to address the concern about spatial regularization and potential overfitting when combining all 13 channels into a single feature vector on a small dataset. A frontal DE gate was tested using only the five prefrontal channels, Fp1, Fp2, F3, Fz, and F4, across the same three frequency bands which gives a 15-dimensional feature vector. These channels were chosen because they cover the prefrontal working memory network and are the primary sites of frontal theta elevation under arithmetic load. Reducing the input from 39 to 15 dimensions also directly reduces the risk of overfitting by cutting the feature-to-subject ratio. The frontal gate was evaluated under the same nested LOO scheme and compared statistically against the full 13-channel DE gate using pairwise Wilcoxon signed-rank tests across *N* = 13 subjects.

## 4 Results

### 4.1 Preprocessing

All 13 subjects completed the full preprocessing pipeline and no trial data was lost at any stage. All 40 trials per part were kept for every subject, giving perfectly balanced Left and Right class distributions of 20 trials per class. Eight subjects had one ICA component removed: S02, S03, S05, S06, S07, S08, S12, and S13. For each of these subjects, the removed component showed the typical frontal distribution and blink-related time course associated with ocular artefacts. The remaining five subjects, S01, S04, S09, S10, and S11, had no component cross the 3.0 SD correlation threshold, so their signals were kept as recorded. Every subject contributed 40 epochs each to Part 3 and Part 4, with exactly 20 Left and 20 Right trials in both parts.

### 4.2 Model selection

Table 1 summarizes Criterion A and Criterion B balanced accuracy for all nine models on pure motor imagery trials and dual-task trials, averaged across all 13 subjects.

**Table 1:** Mean balanced accuracy for all nine classifier pipelines on pure MI and dual-task.

Model	Family	Mean of Criterion A (trained and tested on Part 3)	Mean of Criterion B (trained on Part 3 and tested on Part 4)
FBCSP + SVM-Linear	FBCSP	0.5942	0.5173
FBCSP + SVM-RBF	FBCSP	0.5885	0.5058
FBCSP + LDA	FBCSP	0.5731	0.5288
FBCSP + LR	FBCSP	0.5712	0.5231
MDM	Riemannian	0.5135	0.5154
TSM + LDA	Riemannian	0.5038	0.5250
TSM + SVM-Linear	Riemannian	0.5038	0.5154
TSM + LR	Riemannian	0.5000	0.4981
TSM + SVM-RBF	Riemannian	0.4769	0.5077

**Figure 2:**
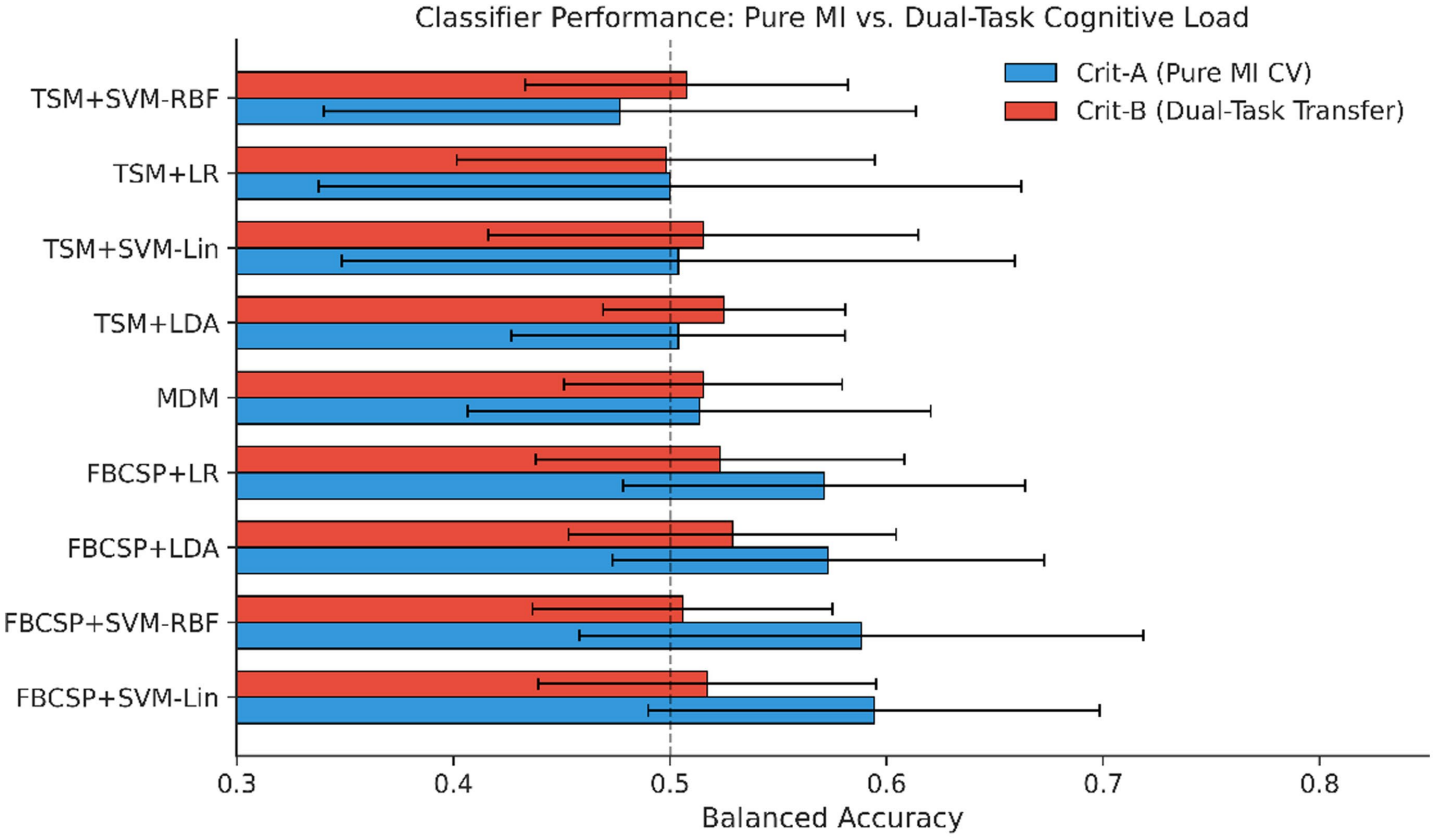
Mean balanced accuracy of all nine classifier pipelines on pure MI (Criterion A) and dual-task (Criterion B) trials, averaged across 13 subjects.

FBCSP + SVM-Linear had the highest Criterion A mean balanced accuracy at 0.594 ± 0.104 and was chosen as the global decoder. All four top Criterion A positions were taken by FBCSP variants, which is explained by the data augmentation that comes from sliding windows, as discussed in Section V. On Criterion B, the two families were much closer to each other. FBCSP + LDA came out as the best individual model on Criterion B at 0.529. The Riemannian methods as a group degraded less from Criterion A to Criterion B than the FBCSP models, but no single Riemannian model crossed 0.529. This is also shown in Figure 2.

### 4.3 Subject-level pure MI performance

Table 2 reports individual balanced accuracies for FBCSP + SVM-Linear on pure motor imagery (Criterion A) and dual-task (Criterion B). Criterion A is the 10-fold stratified cross-validated balanced accuracy on Part 3 trials. Criterion B is the balanced accuracy obtained by training on all 40 Part 3 trials and testing on all 40 Part 4 trials as a batch, with no Part 4 data in the training set. In the gate stage, a LOO scheme runs over Part 4 trials and the remaining 39 Part 4 trials are pooled with the full Part 3 set for each training fold. So the gate-stage predictions have access to Part 4 data that the Criterion B batch evaluation deliberately excludes, for two purposes: Table 2 quantifies how much the decoder degrades moving from pure to dual-task conditions, while the gate stage uses the larger LOO training pool to get the best possible predictions for gate input.

**Table 2:** Per-subject balanced accuracy for FBCSP + SVM-linear on pure MI and dual-task.

Subject	Part 3 Pure MI (Criteria A)	Part 4 dual-task (Criteria B)	Deterioration
S01	0.625	0.525	0.100
S02	0.5	0.525	−0.025
S03	0.775	0.725	0.050
S04	0.5	0.45	0.05
S05	0.6	0.525	0.075
S06	0.575	0.5	0.075
S07	0.525	0.45	0.075
S08	0.775	0.425	0.35
S09	0.425	0.55	−0.125
S10	0.6	0.475	0.125
S11	0.525	0.575	−0.05
S12	0.625	0.55	0.075
S13	0.675	0.45	0.225

**Figure 3:**
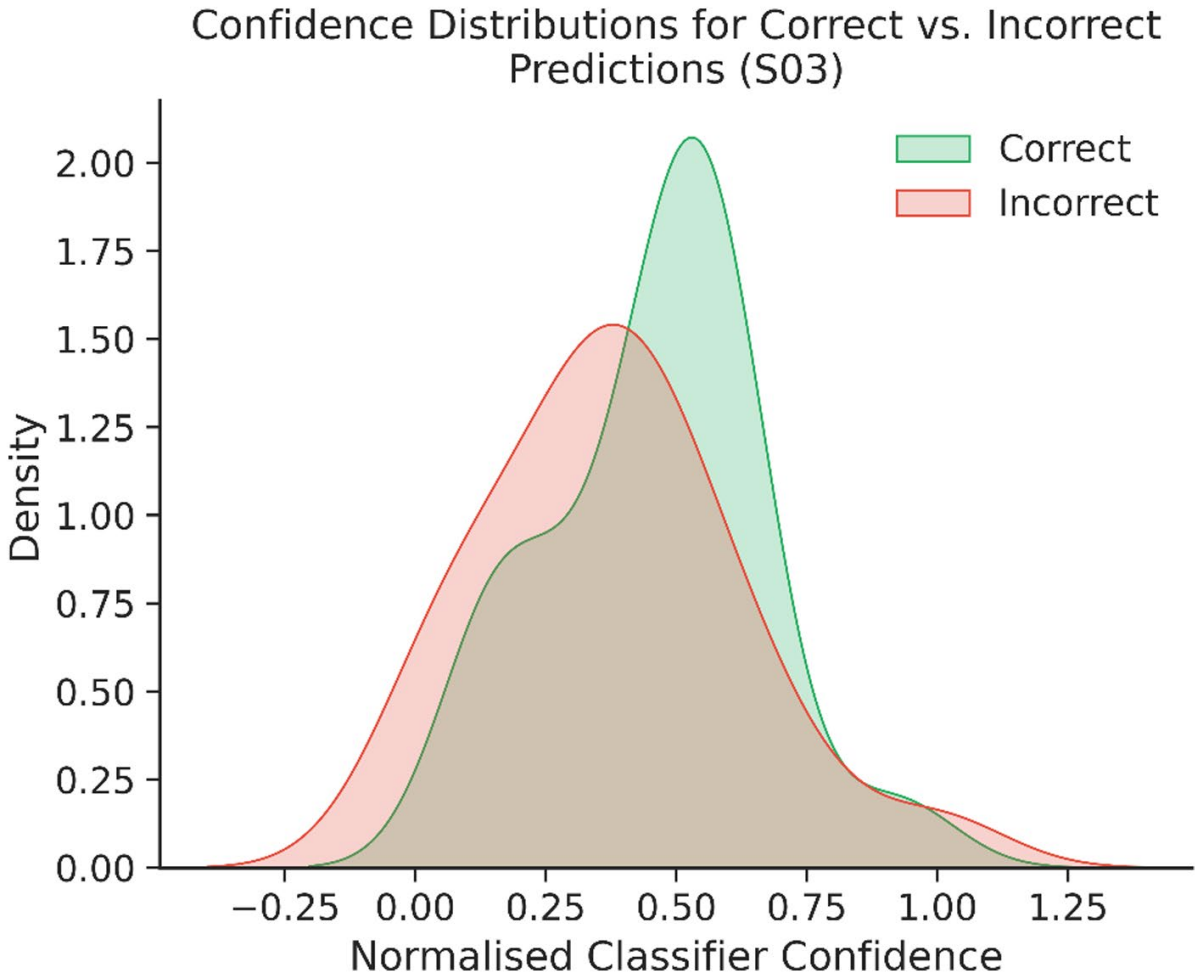
Normalized confidence scores for correct and incorrect predictions on Part 4 dual-task trials, shown for S03, the best-performing subject.

Table 3 lists the individual Criterion A and Criterion B balanced accuracies for FBCSP + SVM-Linear across all 13 subjects. The group mean for Criterion A was 0.594 ± 0.104 and for Criterion B it was 0.517 ± 0.078. A Wilcoxon signed-rank test on the 13 paired values gave W = 14.0, *p* = 0.026, confirming that the drop in MI accuracy when arithmetic is added is statistically significant at the group level. The normalized FBCSP + SVM-Linear model’s decision-margin confidence scores for correct and incorrect predictions on Part 4 dual-task trials for the top-performing subject (S03) is shown in Figure 3.

**Table 3:** Per-subject binomial chance-level test (one-sided, *n* = 40 trials, H0: balanced accuracy ≤ 0.5).

Subject	Crit-A balanced accuracy	Crit-A p	Above chance (A)	Crit-B balanced accuracy	Crit-B p	Above chance (B)
S01	0.625	0.077	No	0.525	0.437	No
S02	0.500	0.563	No	0.525	0.437	No
S03	0.775	<0.001	Yes	0.725	0.003	Yes
S04	0.500	0.563	No	0.450	0.785	No
S05	0.600	0.134	No	0.525	0.437	No
S06	0.575	0.215	No	0.500	0.563	No
S07	0.525	0.437	No	0.450	0.785	No
S08	0.775	<0.001	Yes	0.425	0.866	No
S09	0.425	0.866	No	0.550	0.318	No
S10	0.600	0.134	No	0.475	0.682	No
S11	0.525	0.437	No	0.575	0.215	No
S12	0.625	0.077	No	0.550	0.318	No
S13	0.675	0.019	Yes	0.450	0.785	No

**Figure 4:**
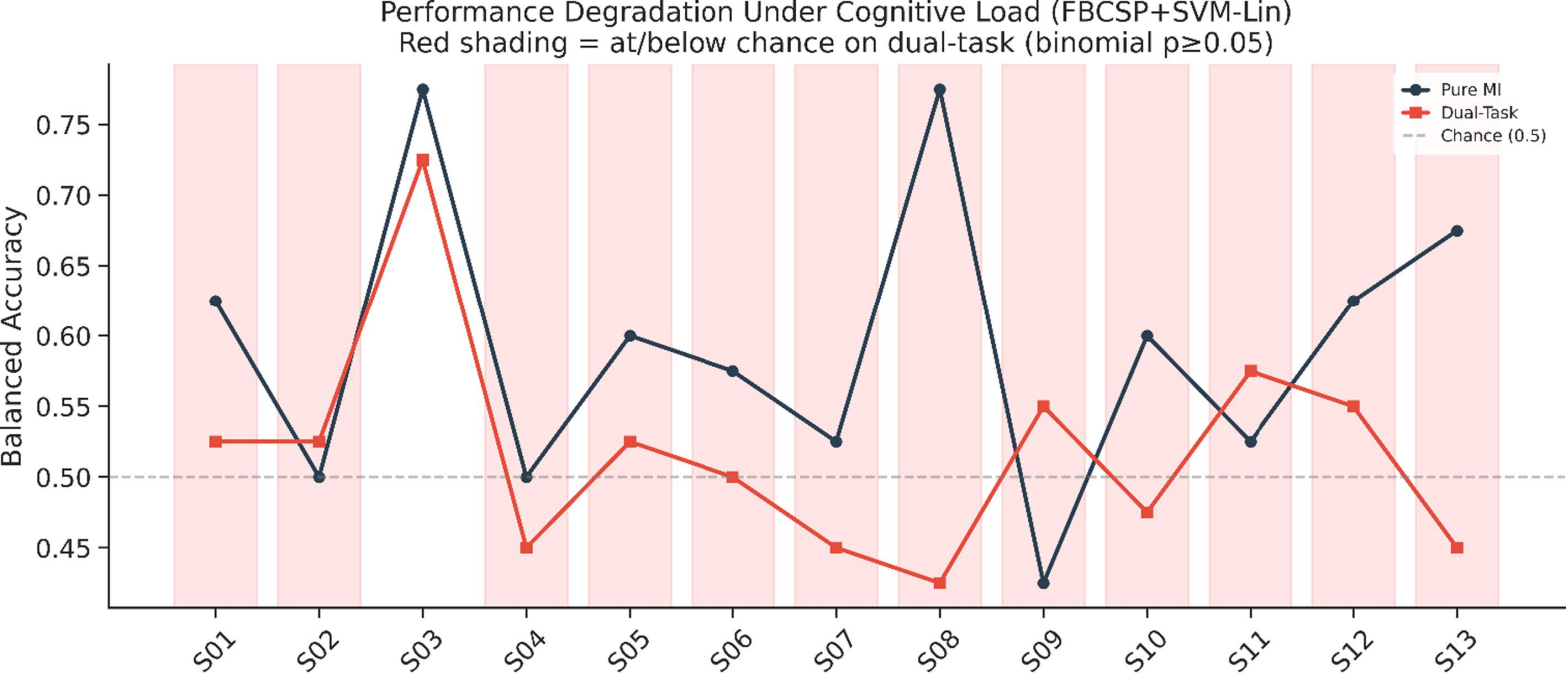
Chance-annotated subject performance: per-subject Criterion A and Criterion B balanced accuracy for FBCSP + SVM-Linear.

A one-sided binomial test with *n* = 40 trials and the null hypothesis that balanced accuracy is at or below 0.5 was applied to each subject’s Criterion A and Criterion B scores. On Criterion A, three subjects were above chance: S03 (*p* < 0.001), S08 (*p* < 0.001), and S13 (*p* = 0.019). On Criterion B, only one subject, S03, was above chance (*p* = 0.003). Every other subject was at or below chance on dual-task MI decoding. This result, as illustrated in Figure 4, directly shapes how all gate findings should be read. For the 12 subjects whose dual-task decoding was at or below chance, the gate is working with predictions that have very little reliable signal in them. The gate results for those subjects show how the system behaves under conditions resembling BCI illiteracy, rather than under functional MI control. A decoder operating above chance is a prerequisite for a gate to perform selective reliability estimation in a meaningful way.

### 4.4 Accuracy by difficulty level

**Figure 5:**
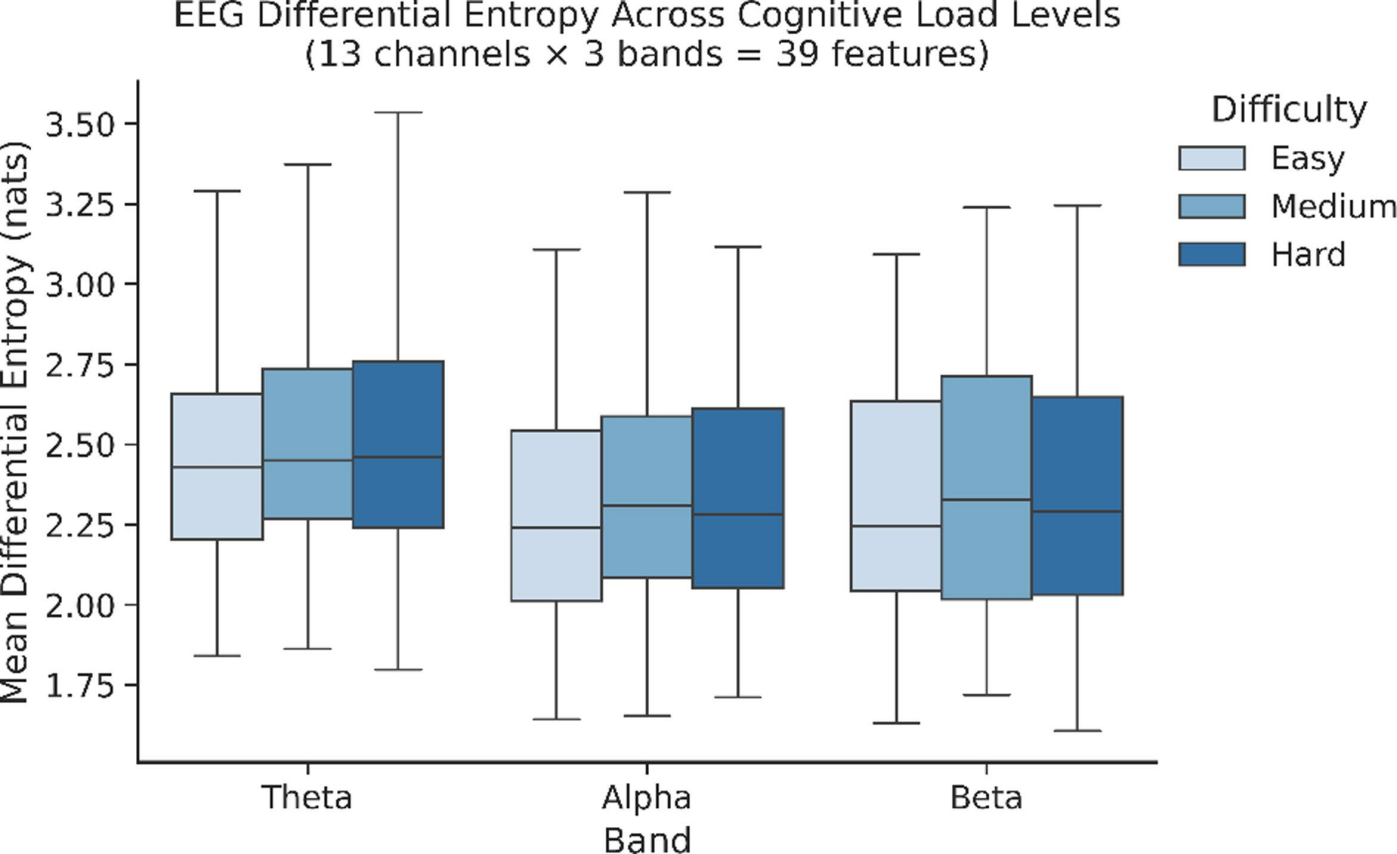
Mean DE feature magnitude across the three concurrent-arithmetic difficulty levels (Easy, Medium, Hard).

Table 4 shows Part 4 MI accuracy broken down by the difficulty of the concurrent arithmetic task. At the group level, accuracy went from 0.500 for Easy to 0.481 for Medium and 0.452 for Hard. A Friedman test across the three difficulty levels, treating per-subject accuracy as the repeated measure, gave χ^2^ = 0.280, *p* = 0.869. This means the difficulty effect is not statistically significant. The monotonic drop seen in the group averages should be read as an exploratory trend that is in line with the cognitive load hypothesis, but it is not a confirmed finding. There were roughly 12 trials per subject for Easy and Medium and about 16 for Hard. At those trial counts, within-subject variance is simply too large for a reliable within-subject test. Larger trial counts per difficulty level will be needed to properly test whether graded cognitive load produces a graded degradation in MI accuracy (Figure 5).

**Table 4:** MI classification accuracy on Part 4 dual-task trials grouped by concurrent arithmetic difficulty.

Difficulty	Mean accuracy	N trials
Easy	0.500	156
Medium	0.481	156
Hard	0.452	208

### 4.5 Gate performance

**Figure 6:**
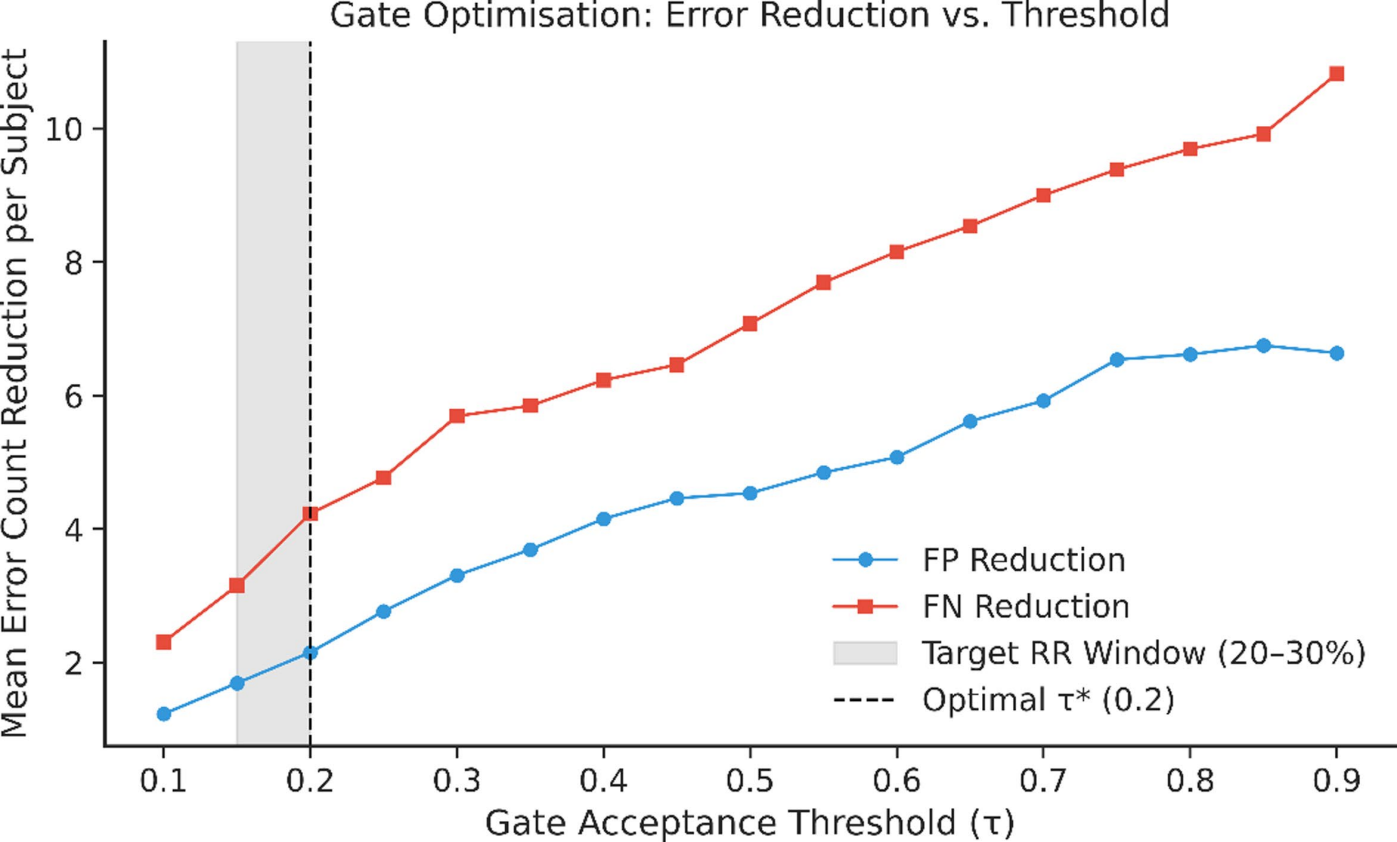
Threshold sweep curve: FP and FN reduction vs. *τ*, with the target RR window shaded and τ* = 0.20 marked.

**Figure 7:**
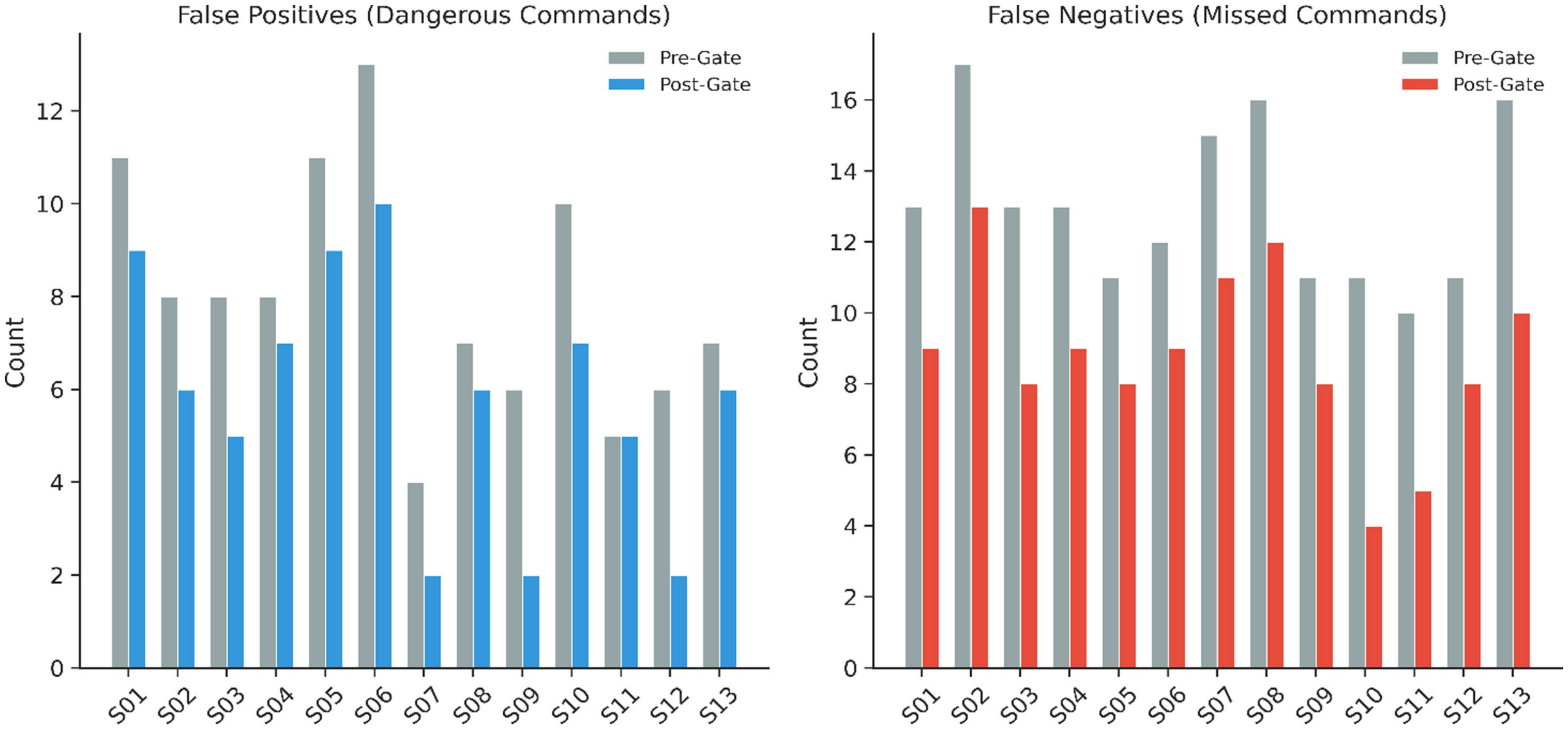
Per-subject false positive (left panel) and false negative (right panel) command counts before (pre-gate) and after (post-gate) cognitive load gate application, for all 13 subjects.

**Figure 8:**
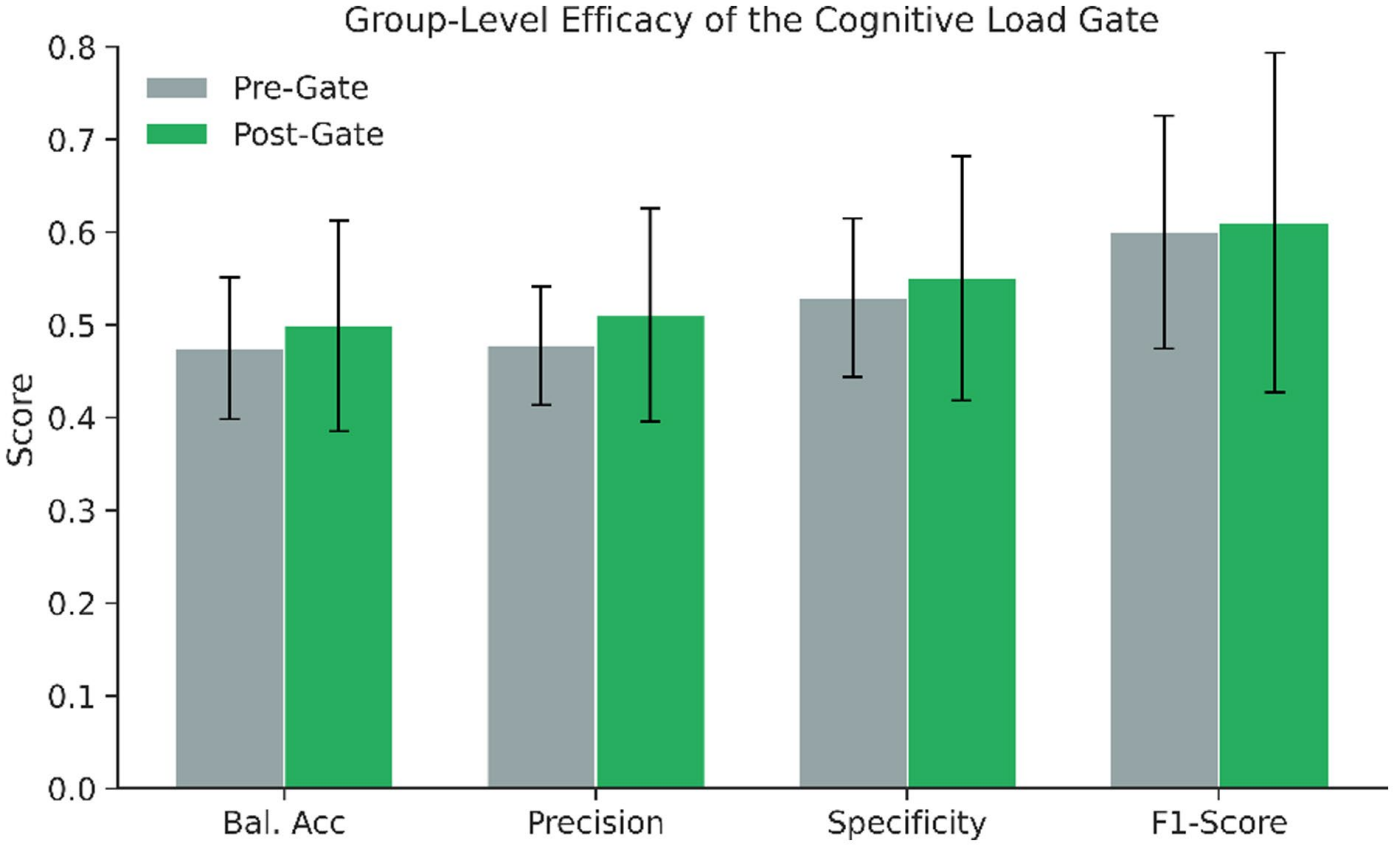
Mean balanced accuracy, precision, specificity and F1 score pre-gate and post-gate cognitive load gate application, averaged across 13 subjects.

**Figure 9:**
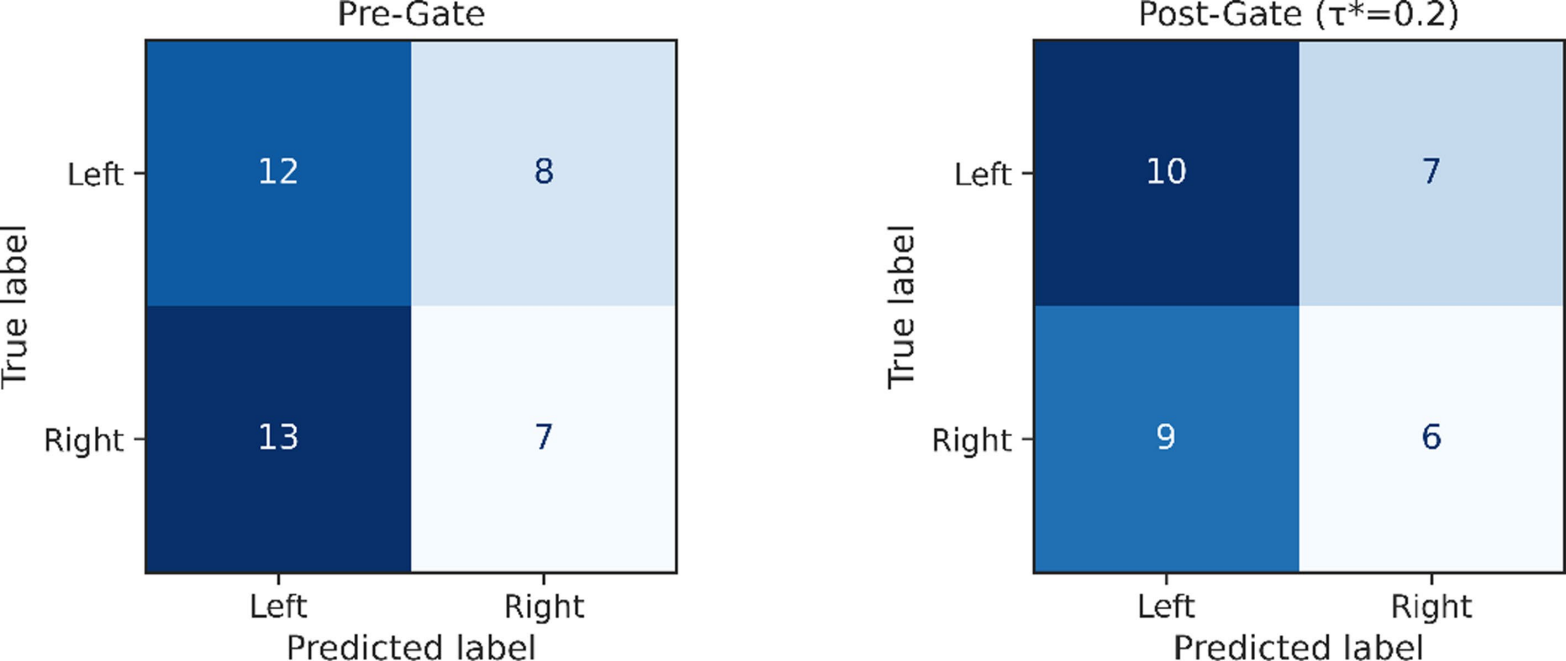
Confusion matrix for FBCSP + SVM-Linear predictions on Part 4 dual-task trials from S03, the best-performing, shown pre- and post-gate.

**Figure 10:**
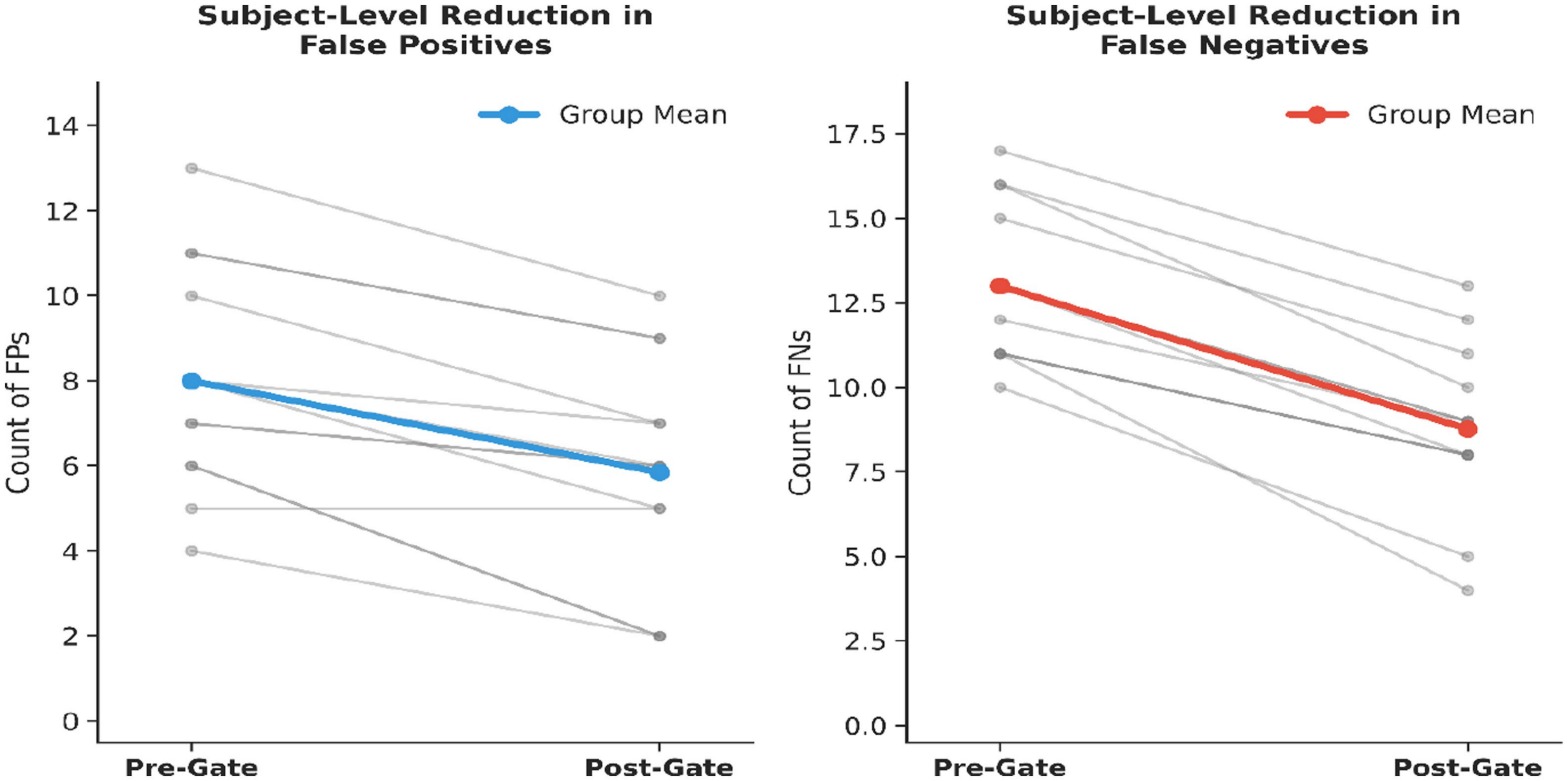
Subject-specific reductions in false positives (left) and false negatives (right) following gate application. Gray transparent lines represent individual subjects, and the bold lines depict the group mean.

Table 5 shows the full pre-gate and post-gate comparison across all 13 subjects under the nested LOO scheme. The combined gate ran at a mean rejection rate of 25.0%, which sits within the 20–30% target window (Ferrez and Millán, 2008). Balanced accuracy improved significantly post-gate (+0.031, *p* = 0.013) and F1 showed a trend in the same direction that did not quite reach significance (+0.033, *p* = 0.080). False positive commands dropped from 8.00 to 5.62 per subject (*Δ* = −2.38, *p* < 0.001) and false negative commands from 13.00 to 9.00 per subject (Δ = −4.00, *p* < 0.001). Precision also improved significantly (+0.036, *p* = 0.013). Recall and specificity both moved in the positive direction but neither reached significance (*p* = 0.223 each). Figure 6 shows the threshold sweep curve and Figures 7–10 illustrate the pre-gate and post-gate performance metrics.

**Table 5:** Pre- and post-gate performance metrics averaged across *N* = 13 subjects (nested LOO).

Metric	Pre-gate	Post-gate	Difference	*p*-value
Balanced accuracy	0.475 ± 0.077	0.506 ± 0.105	+0.031	0.013
Precision	0.478 ± 0.064	0.514 ± 0.094	+0.036	0.013
Recall (true positive rate)	0.350 ± 0.116	0.383 ± 0.155	+0.033	0.223
Specificity (true negative rate)	0.600 ± 0.131	0.633 ± 0.177	+0.033	0.223
F1 score	0.394 ± 0.111	0.427 ± 0.142	+0.033	0.080
False positives	**8.000 ± 2.512**	**5.615 ± 2.528**	**−2.385**	**<0.001** ^**a**^
False negatives	**13.000 ± 2.219**	**9.000 ± 2.000**	**−4.000**	**<0.001** ^**a**^
Rejection rate	0.000	0.250 ± 0.048	+0.250	**<0.001** ^**a**^

^a^Statistically significant at *p* < 0.05, two-tailed Wilcoxon signed-rank test, *N* = 13 subjects. Bold values indicate statistically significant differences between pre-gate and post-gate metrics (*p* < 0.05, two-tailed Wilcoxon signed-rank test).

A note on the recall and specificity values: both are computed directly from confusion counts, recall as TP/(TP + FN) and specificity as TN/(TN + FP). The pre-gate group means are different at 0.350 for recall and 0.600 for specificity. This gap exists because even though the trial counts are balanced (20 Left and 20 Right per subject), the classifier makes more Left-classified-as-Right errors (high FN) than Right-classified-as-Left errors (FP). So recall is lower than specificity at the group level.

**Figure 11:**
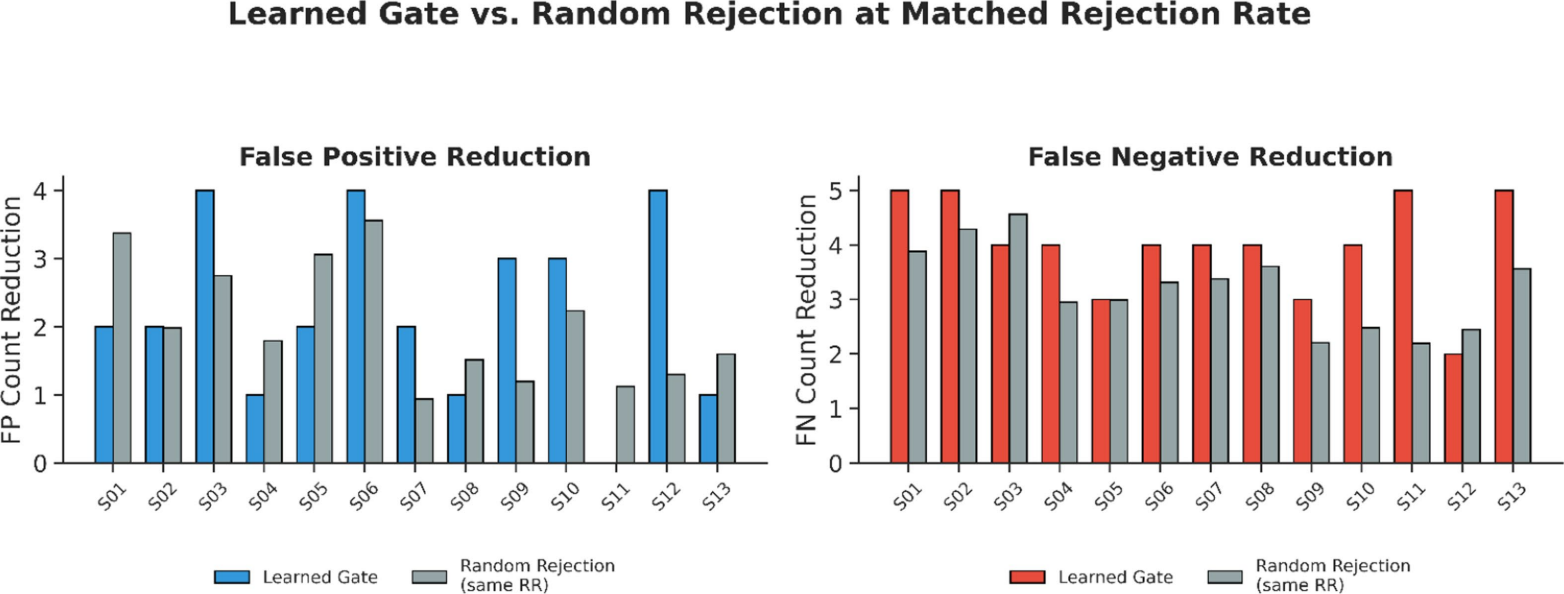
Random baseline comparison: learned gate FP and FN reductions compared against matched random rejection baseline across 13 subjects.

**Figure 12:**
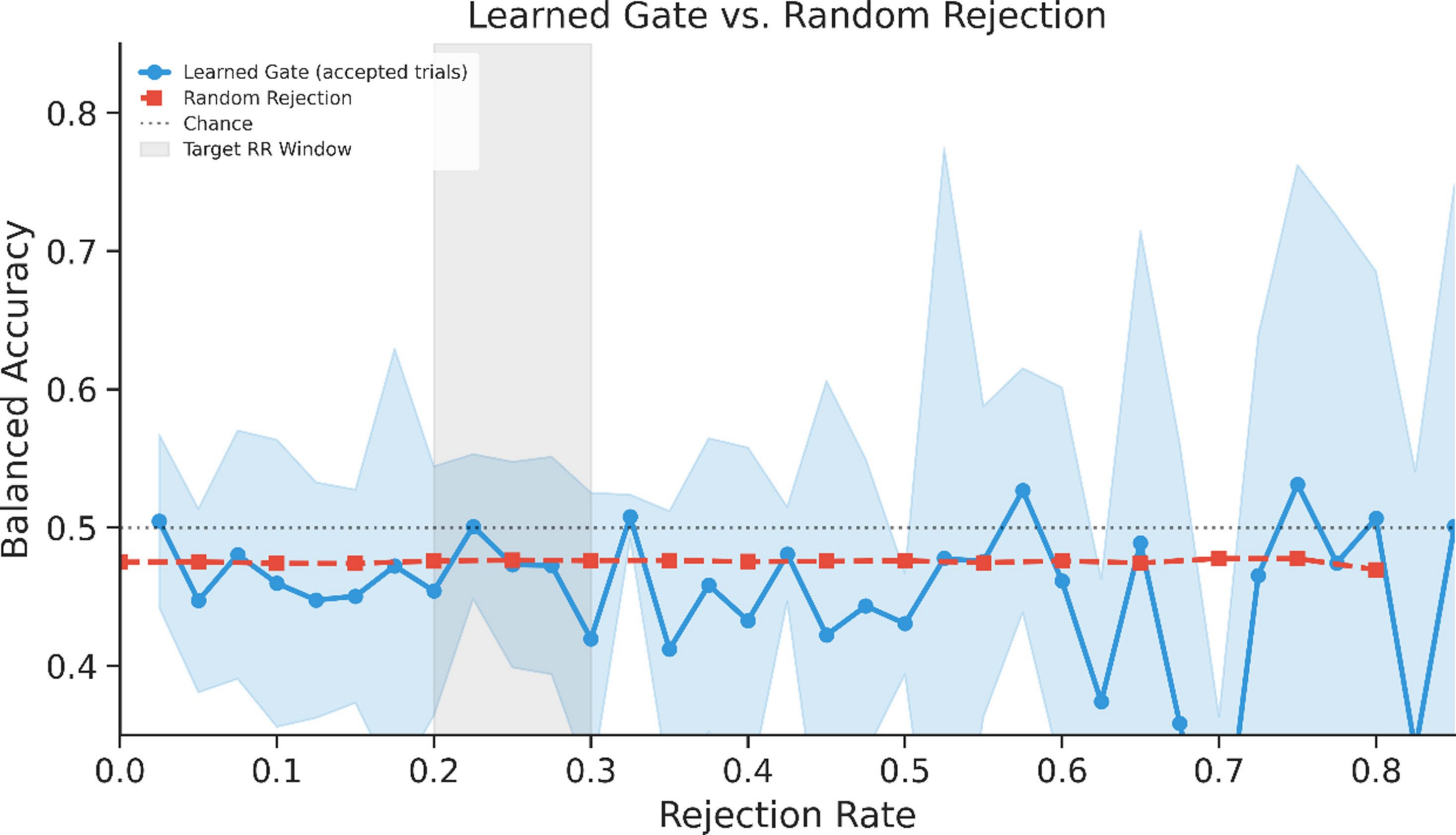
Mean balanced accuracy of accepted trials as a function of rejection rate, comparing the learned cognitive-load gate (blue) against matched random rejection (red dashed) under the nested LOO scheme, averaged across 13 subjects. The shaded band shows ±1 SD across subjects for the learned gate, the dotted line marks chance-level accuracy (0.50) and the grey band marks the 20–30% target rejection-rate window.

To check whether the gate’s error reductions go beyond what simply withholding a fraction of trials at random would give, a permutation baseline was computed. For each subject, 1,000 random subsets of the same size as the gate-accepted set were drawn, and mean FP and FN reductions were computed per permutation. At the group level, the gate’s FN reductions were significantly higher than the random baseline mean (Wilcoxon *p* = 0.006), but FP reductions were not (*p* = 0.455). At the individual subject level, only 2 of 13 subjects exceeded the 95th percentile of the random distribution on FP reduction, and only 1 of 13 did on FN reduction. The gate therefore shows selective FN suppression that goes beyond random rejection, but the FP suppression result cannot be distinguished from what random withholding would produce at the same rate. Figures 11, 12 show the results of the same.

### 4.6 Ablation results

Table 6 gives the group-level performance of all three gate variants under the same nested LOO scheme.

**Table 6:** Ablation study: comparison of three gate variants.

Gate	Mean FP reduction	Mean FN reduction	Mean rejection rate	*p*-value compared to combined gate (FP)	*p*-value compared to combined gate (FN)
DE + Confidence (Combined)	2.385	4.000	25.0%	—	—
DE Only	2.231	3.615	23.5%	0.688	0.325
Confidence Only	1.000	2.077	12.3%	**0.037** ^**a**^	**0.030** ^**a**^

^a^Statistically significant, two-tailed Wilcoxon signed-rank test, *N* = 13 subjects. Bold *p*-values indicate a statistically significant difference from the combined (DE+Confidence) gate.

The confidence-only gate ran at a mean rejection rate of just 12.3%, which is well below the 20–30% target window. It produced a mean FP reduction of 1.000 per subject and FN reduction of 2.077 per subject, both of which were significantly lower than the fused gate (FP: *p* = 0.037, FN: *p* = 0.030). The DE-only gate gave mean FP reduction of 2.231 and FN reduction of 3.615 at a rejection rate of 23.5%, and neither of those values differed significantly from the fused gate (FP: *p* = 0.688, FN: *p* = 0.325). Therefore, DE features are what drive the gate and that including the confidence score does not add anything statistically significant on top of that.

Table 7 shows the channel subset comparison with two separate statistical tests reported for each gate variant: whether its FP and FN reductions are significant against the pre-gate baseline, and whether it differs from the full 13-channel gate. The full 13-channel DE gate produced mean FP reduction of 2.231 per subject and FN reduction of 3.615 per subject at a rejection rate of 23.5%, with both reductions significant against the pre-gate baseline (FP: *p* < 0.001, FN: *p* < 0.001). The frontal-only DE gate, built from just the five prefrontal channels Fp1, Fp2, F3, Fz, and F4 with a 15-dimensional feature vector, gave mean FP reduction of 2.154 per subject and FN reduction of 3.692 per subject at a rejection rate of 24.2%, also significant against the pre-gate baseline (FP: *p* < 0.001, FN: *p* < 0.001). When the two gates were compared directly against each other, there was no significant difference on FP reduction (*p* = 1.000) or FN reduction (*p* = 0.813). The frontal gate additionally produced significant post-gate improvements in precision (*p* = 0.022) and F1 (*p* = 0.040). Both gates are significant against the no-gate baseline, and neither is significantly better than the other, confirming that the 39-dimensional full gate is not providing any measurable benefit over a much simpler 15-dimensional frontal gate in this dataset. The frontal gate also gave significant post-gate improvements in precision (*p* = 0.022) and F1 (*p* = 0.040). The fact that a 15-dimensional frontal gate matches the full 39-dimensional gate confirms that the full gate is not overfitting to the input space on this *N* = 13 dataset, and that frontal channels carry essentially all of the information the gate needs.

**Table 7:** Channel subset comparison: baseline significance and frontal-channel vs. full-channel comparison.

Gate variant	Mean FP reduction	Mean FN reduction	Mean rejection rate	p-value compared to pre-gate baseline (FP)	p-value compared to pre-gate baseline (FN)	p-value compared to 13-channel gate (FP)	p-value compared to 13-channel gate (FN)
13 channel, 39-dimensional DE gate	2.231	3.615	23.5%	**<0.001** ^**a**^	**<0.001** ^**a**^	—	—
5 channel, 15-dimensional frontal gate	2.154	3.692	24.2%	**<0.001** ^**a**^	**<0.001** ^**a**^	1.000	0.813

^a^Two-tailed Wilcoxon signed-rank test against pre-gate counts, *N* = 13 subjects. Bold *p*-values indicate statistical significance (*p* < 0.05, two-tailed Wilcoxon signed-rank test against the pre-gate baseline counts).

## 5 Discussion

### 5.1 Low overall accuracy

The cross-subject mean Criterion A balanced accuracy of 0.594 falls within the 0.55–0.65 range that is commonly reported for BCI-naive subjects on moderate-density montages (Lotte et al., 2018). However, the per-subject binomial test reveals a more important picture. On Criterion A, only 3 of 13 subjects, S03, S08, and S13, were above chance. On Criterion B, which is the condition the gate is actually applied to, only S03 was above chance. For the remaining 12 subjects on Criterion B, the decoder’s predictions do not carry enough reliable information for a gate to meaningfully pick out which trials are correct and which are not. The gate results for those subjects reflect what happens when a cognitive load gate is run on top of a near-chance decoder, which is different from a functional MI decoding scenario. The FP and FN reductions at the group level are statistically significant relative to the pre-gate baseline, but they need to be read with that context in mind. The fact that FN reductions exceeded random rejection while FP reductions did not is consistent with this picture. Despite near-chance decoding in 12 of 13 naive participants, the DE-based gate still recovered a statistically robust, above-random reduction in false-negative commands, suggesting the gate is picking up genuine cognitive-load information independent of decoder quality. Selective FP suppression is harder to achieve when the decoder itself largely produces noise.

All 13 participants were neurologically naive with no prior BCI training. BCI-naive participants typically require multiple calibration sessions before ERD/ERS patterns reach the consistency that CSP and Riemannian methods can exploit (Sannelli et al., 2019). Competition datasets involve participants who received explicit neurofeedback training beforehand, and their decoding accuracies should not serve as benchmarks for naive populations.

Second, the 0.5-s cue-response buffer means that only 3.5 s of imagery signal are used per trial. It is also possible that some subjects simply took longer than 0.5 s to start imagining after reading the cue word. A future study including a brief MI training session before recording would likely increase baseline accuracy significantly.

### 5.2 Better performance of FBCSP

The established literature result of Riemannian methods outperforming FBCSP on small samples was not replicated in Criterion A. Each 3.5-s trial produced roughly 6 overlapping 1-s windows under the FBCSP, and majority vote collapsed those back to a single trial prediction. In effect, FBCSP saw six times as many training examples per fold as the Riemannian methods did, which fed the downstream SVM or LDA considerably more data. The Riemannian pipeline worked with one covariance matrix per trial, without any augmentation or windowing. In a 10-fold scheme with 40 trials per subject, this asymmetry explains FBCSP’s advantage in Criterion A.

The Criterion B numbers in Table 1 do not support the claim that Riemannian methods outperform FBCSP on cross-condition transfer. FBCSP + LDA had the highest individual Criterion B accuracy at 0.529. MDM reached 0.515 and TSM + LDA 0.525, both of which are lower than FBCSP + LDA. The Riemannian family as a whole did show less average degradation from Criterion A to Criterion B than the FBCSP family, which is consistent with the theoretical expectation that covariance-based methods are less sensitive to spatial non-stationarity (Miao et al., 2021). However, at the level of individual model comparisons, no Riemannian model beat the best FBCSP model on transfer.

### 5.3 Accuracy of graded mental arithmetic

The group-level accuracy went in the expected direction, from 0.500 at Easy down to 0.481 at Medium and 0.452 at Hard. The Friedman test across these three levels gave χ^2^ = 0.280, *p* = 0.869, which is not statistically significant. So the monotonic trend in the group means is an exploratory observation, not a confirmed finding. At around 12 trials per subject for Easy and Medium and about 16 for Hard, the per-subject accuracy estimates at each difficulty level are too noisy for reliable individual-level analysis. Several subjects showed non-monotonic patterns at the individual level, which is consistent with this level of within-subject variance. The group mean showing a monotonic drop is an averaging effect rather than evidence of a true graded relationship. Replication with more trials per difficulty level is needed before this can be treated as a confirmed result.

### 5.4 Gate performance and error reductions

The gate gave statistically significant reductions in FP (*Δ* = −2.385, *p* < 0.001) and FN (Δ = −4.000, *p* < 0.001), along with significant gains in balanced accuracy (+0.031, *p* = 0.013) and precision (+0.036, *p* = 0.013). All accuracy metrics moved in the positive direction post-gate, which is consistent with the gate removing unreliable predictions, though the result has to be read carefully given that 12 of 13 subjects were at or below chance on Criterion B.

The random baseline comparison puts the FP and FN reductions into perspective. FN reductions from the learned gate were significantly larger than what random rejection produced at the same rate (Wilcoxon *p* = 0.006). FP reductions were not (*p* = 0.455). In wheelchair terms, the FN benefit means the gate is reducing commands that would send the chair the wrong way; the FP null result is discussed further in Section VI.

The ablation results show that DE features do the work. The confidence-only gate did not reach the target rejection window for most subjects and gave significantly weaker FP and FN reductions than the fused gate (*p* = 0.037 and *p* = 0.030). The DE-only gate matched the fused gate on both (FP: *p* = 0.688, FN: *p* = 0.325). Adding the confidence score on top of DE does not change the outcome in any statistically measurable way. A DE-only gate is therefore sufficient and the confidence score adds no measurable benefit. The frontal 15-dimensional gate matched the full 39-dimensional gate, which confirms that frontal theta channels carry essentially all the relevant information and that the broader channel set is not needed.

### 5.5 Clinical and safety interpretation

This clinical interpretation is based on an offline study of 13 healthy young adult participants with no motor impairments. These results do not establish clinical safety for the target population of individuals with ALS, spinal cord injury, or stroke, who exhibit different neurophysiological signatures, altered cortical reorganization, and different fatigue profiles. The findings represent a proof-of-concept demonstration whose applicability to patient populations must be validated separately.

The clinical reading of these results needs to be done carefully. Only 1 of 13 subjects had above-chance MI decoding on dual-task trials. For the other 12, the decoder’s outputs are statistically indistinguishable from random, which means the gate is not performing selective error suppression in the usual sense for those subjects. The actual gate benefit is narrower than the raw FP and FN numbers might suggest: FN suppression is selective and exceeds random rejection, but FP suppression is not. In a real deployment under near-chance decoding, the gate’s main contribution would be to reject high-cognitive-load trials rather than to specifically identify error-prone ones.

From a safety standpoint in wheelchair use, both the FP and FN reductions still translate to something concrete. Each unit of FP reduction is one fewer unintended movement per session, and each unit of FN reduction is one fewer misclassified command. The mean rejection rate of 25.0% means about one in four commands gets held back, which stays within the target range that is considered clinically workable (Ferrez and Millán, 2008). When a command is withheld, the user tries again, which adds around 4–6 s in a slow indoor navigation setting (Lim et al., 2018). S03 was above at 35.0% and S09 was below at 17.5%. The higher rate for S03, who was the best-performing subject in this study, is consistent with that subject’s gate having learned a sharper decision boundary and is not a sign that the system is misbehaving.

## 6 Limitations

The most important finding from the chance-level analysis is that only 1 of 13 subjects showed statistically above-chance MI decoding under dual-task conditions. This limits what the gate can realistically do, because reliable sorting of correct and incorrect predictions requires the predictions themselves to carry some useful information. The fact that the gate’s FN reductions exceed random rejection despite this shows that DE features do contain information about which trials are high-load, even when the decoder is near chance. However, the FP result does not rise above random, which is the expected outcome when the underlying predictions are largely uninformative. These are limitations that come from the choice of a healthy, naive, untrained participant sample. Both of them could be partially addressed by giving participants MI training sessions before recording, which has been shown to improve accuracy by roughly 10–15 percentage points in similar populations (Vidaurre et al., 2011), and by recruiting participants who already have some BCI exposure.

This study is entirely offline. EEG was recorded and analysed after the session, with no real-time feedback given to participants and no wheelchair connected. Moving to a live system introduces challenges that the current work cannot address.

On the computational side, the gate must produce an accept or reject decision within the 500 ms inter-window step. DE computation takes roughly 0.3 ms per channel on standard hardware, and logistic regression inference adds negligible time, so meeting the timing requirement is realistic. The bigger open question is how the system feels to use. When a command is withheld, the user needs to notice it, recompose the motor imagery, and try again. Whether this is disruptive depends on how often it happens and how long the retry takes. This can only be studied with an online experiment that gives participants real feedback.

The gate was trained and tested within a single session per subject. EEG-based cognitive load markers are not stable across sessions (Sadatnejad and Lotte, 2022). A gate trained one day may operate at a different threshold the next because the spectral properties of the signal drift. Vidaurre et al. (2011) showed exactly this for LDA classifiers fitted on one BCI session and applied to a later one, which is part of what motivated their unsupervised adaptation work. Future studies should test multi-session recordings and check whether the gate’s performance holds up, and if it does not, how little data is needed at the start of a new session to recalibrate it. The long-term adaptive shared-control framework used by Saeedi et al. (2017) over eleven months is a useful reference point for how this could be designed.

No MI training was given to participants before recording. All 13 subjects were neurologically naive, and it is well established that naive participants need multiple neurofeedback-guided MI sessions before their ERD patterns become consistent enough for classifiers to exploit reliably (Sannelli et al., 2019). Two to three training sessions before data collection have been shown to raise accuracy by ten to fifteen percentage points in comparable populations. Better baseline decoding would give the gate a more informative signal to work with.

The accuracy difference across difficulty levels (Easy: 0.500, Medium: 0.481, Hard: 0.452) spans only 0.048 units. The direction is right but the range is narrow. Each difficulty level contributes around 12 trials per subject for Easy and Medium, and about 16 for Hard. At those counts, within-subject variance is large enough to obscure the trend at the individual level, and only the group average brings it out. A version of this study with more trials per difficulty level would allow proper statistical testing of the graded degradation effect rather than just reporting means.

The channel subset analysis showed that a frontal DE gate performs equivalently to the full 13-channel version in this study. While this is a useful finding, it is specific to a mental arithmetic load manipulation where frontal theta is the dominant EEG response. Other cognitive load tasks, such as spatial reasoning or emotional stress, may recruit parietal, temporal, or occipital regions more strongly. Whether the frontal-only gate generalizes across different secondary task types has not been tested here and remains an open question.

All participants were healthy young adults between 17 and 27 years old with no motor impairments. The intended clinical users are people with ALS, spinal cord injury, or brainstem stroke, but they all have different neural profiles, different fatigue characteristics, and in many cases, altered cortical organisation from disease or long-term disuse. The current results do not establish that the gate generalizes to those populations. This work should be understood as an early offline proof-of-concept, not as a clinically validated safety framework.

## 7 Conclusion

A wheelchair-driving BCI based on motor imagery has safety requirements that go well beyond what accuracy numbers alone can tell us. This study showed that adding simultaneous mental arithmetic caused a statistically significant group-level drop in MI decoding accuracy (Criterion A: 0.594, Criterion B: 0.517, W = 14.0, *p* = 0.026). However, per-subject binomial tests showed that only 1 of 13 subjects was above chance under dual-task conditions, and a Friedman test found no statistically significant effect of arithmetic difficulty on MI accuracy (χ^2^ = 0.280, *p* = 0.869). Both of these are exploratory-level observations rather than confirmed findings.

The DE-based safety gate gave significant reductions in false positives (−2.385 per subject, *p* < 0.001) and false negatives (−4.000 per subject, *p* < 0.001), with significant improvements in balanced accuracy (*p* = 0.013) and precision (*p* = 0.013). The FN reductions were significantly larger than matched random rejection (*p* = 0.006), which shows selective FN suppression. FP reductions did not exceed random rejection (*p* = 0.455). The ablation study showed that the confidence-only gate underperformed significantly (FP: *p* = 0.037, FN: *p* = 0.030) while the DE-only gate matched the combined gate. A 15-dimensional frontal DE gate using only five prefrontal channels also matched the full 39-dimensional gate, which means a simpler frontal-theta approach is enough for this type of dual-task design.

FBCSP + SVM-Linear had the best Criterion A accuracy and FBCSP + LDA had the best individual Criterion B accuracy. No Riemannian model surpassed the best FBCSP model on transfer, which shows that model rankings depend on the evaluation conditions and that single-condition cross-validation is not enough for choosing models in dual-task BCI settings.

All participants were healthy young adults with no motor impairments. These results should be read as a preliminary offline proof-of-concept. Getting above-chance decoding in more participants, running the system online, giving real-time feedback, and testing with actual patient populations are all necessary before any clinical use.

## Data Availability

The raw data supporting the conclusions of this article will be made available by the authors, without undue reservation.
